# Simultaneous laparoscopic partial pericystectomy and cholecystectomy: Is it safe? A case report

**DOI:** 10.1016/j.ijscr.2019.03.028

**Published:** 2019-03-28

**Authors:** Aliaa Bakr, Mhd Belal Alsabek, Abdul Rahman Hammadieh

**Affiliations:** a Syrian Private University, Faculty of Medicine, Damascus, Syria; b Al-Mouwasat University Hospital, Damascus University, Faculty of Medicine, Damascus, Syria

**Keywords:** Case report, Hydatid liver cyst, Laparoscopic hepatic pericystectomy, Combined, Laparoscopic surgery, Simultaneous laparoscopic surgery

## Abstract

**Background:**

Initial reservations regarding spillage and the ability to manage the cyst wall have been allayed by many reports which show satisfying results with minimal complications using laparoscopic techniques. Here, we study the possibility and safety of simultaneous laparoscopic pericystectomy with one of the most common laparoscopic procedure; laparoscopic cholecystectomy.

**Case presentation:**

A 37 y/o female from a rural area in Syria was referred to our clinic after one year of total pericystectomy from both right and left lobes of lung. She was scheduled for a second operation to resect a third hydatid cyst from her liver. We noted that she also complained of a cholelithiasis and it would be very helpful to manage both these problems altogether.

**Discussion:**

previously, one manuscript discussed in details the safety of laparoscopic simultaneous partial pericystectomy and total cystectomy for hydatid liver cysts. The anaphylactic shock, surgical site infection (SSI) and recurrence were still challenges in any participation of laparoscopic hydatid pericystectomy with other kind of surgery from the same ports. Here, we also paid an attention for the duration and cost of this practice.

**Conclusion:**

The combination of the laparoscopic pericystectomy with other producers may be safe in certain selective cases and does not increase the possibility of postsurgical complications like infections or recurrence. We found this intervention could be effective and useful.

## Highlights

• Possibility of laparoscopic approach should be studied in every hydatid cyst case.
• Simultaneous laparoscopic procedure doesn’t increase rate of complications.
• Skills and instruments should be improved continuously.
• This improvement perform safely more complicated laparoscopic procedures.

## 1 Introduction

Human hydatid disease (cystic echinococcosis, CE) is a chronic parasitic infection caused by the larval stage of the cestode Echinococcus granulosus [1]. It is globally distributed in most pastoral and rangeland areas of the world, with highly endemic areas in the eastern part of the Mediterranean region, northern Africa, southern and eastern Europe, at the southern tip of South America, in Central Asia, Siberia and western China. The incidence rates in these areas can exceed 50 per 100 000 person-years; prevalence levels as high as 5–10% may occur in parts of Argentina, Central Asia, China, East Africa and Peru [2].

Syria lies in the endemic area of the Middle East. We received more than 50 cases of liver hydatid cysts annually in our institute, the main hospital in Damascus. Historically, open surgery has been always in the corner of the management. However, Syrian surgeons, like their colleagues in endemic countries, improved their skills in the laparoscopic management of the liver hydatid cyst. This mini-invasive procedure was firstly described by Saglam et al. [3]. Since that time, many case reports and series have been published [4,5]. The outcome and complications of this procedure were then reported in one systematic review [1]; which stated the availability of variant techniques in the laparoscopic field. One manuscript discussed the combination of partial pericystectomy and total cystectomy in one laparoscopic procedure [6]. To the best of our knowledge, there is no previous publication in PubMed Library that cares about the simultaneous laparoscopic pericystectomy and another kind of abdominal surgery. Our case report opens the door for this question and for further studies and researches.

## 2 Case presentation

A 37 y/o female from a rural area in Syria was referred to our clinic after one year of total open thoracic pericystectomy from right and left lobes of lung; the surgical management of hydatid cyst of the liver was delayed till this admission after two shots of albendazole treatment.

The patient recently developed also a symptomatic cholelithiasis. There were no other obvious findings in physical examination. Laboratory investigations were in normal limited [Table 1].

**Table 1:** Some laboratory tests of patient.

WBCs	5.0 × 10^3^ ul	Lym. (32.1 %), Neut.(61.1%)	rr(3.5–10.0) × **10^3^**ul
RBCs	4.53 × 10^6^ul		rr(3.50–5.50) × 10^6^ul
Platelets	231 × 10^3^ul		rr(100–400) × 10^3^ul
SGPT(ALT)	12.5 UL		rr(7–56) UL
a-Amylase	77.4 UL		rr(40–140) UL

**Fig. 1:**
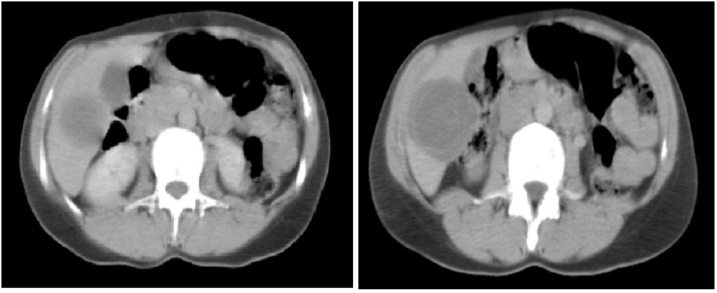
Abdominal CT shows liver hydatid cyst in the VI segment.

The ultrasound showed a cyst in the right lobe of liver and gallbladder stones with no signs of inflammatory. The abdominopelvic computerized tomography (CT) revealed a cystic mass of 5 × 4.5 cm2 with regular borders and minimal calcifications in the 6th segment of liver [Fig. 1]. It was classified as a second degree in the Gharbi classification system [Table 2].

**Table 2:** Gharbi classification of hydatid cysts [9].

Gharbi Classification	
Type I	Pure fluid collection - univesicular cyst.
Type II	Fluid collection with a split wall—detached laminated membrane—“water lily” sign.
Type III	Fluid collection with septa—daughter cysts.
Type IV	Heterogeneous appearance—presence of matrix—mimics a solid mass.
Type V	Reflecting thick walls—calcifications.

The plan of the management was discussed and included lagrot partial pericystectomy and cholecystectomy in the same time. The anatomical location of the hydatid liver cyst allowed to use the same ports for both procedures and it alerted, in the same way, to the risk of facing adhesions in the surgical field if we delayed one procedure to another operation which would be scheduled later. This judgment encouraged our team to run both procedures simultaneously.

## 3 The surgical technique

The laparoscopic surgery was performed. The camera port was applied through the umbilicus following creation of pneumoperitoneum; the hydatid cyst is identified on the lower surface of the liver. Then, the trocar (10 mm) placed in epigastric region and another trocar (5 mm) with cannula is introduced into the peritoneal cavity directly over the hydatid cyst.

**Fig. 2:**
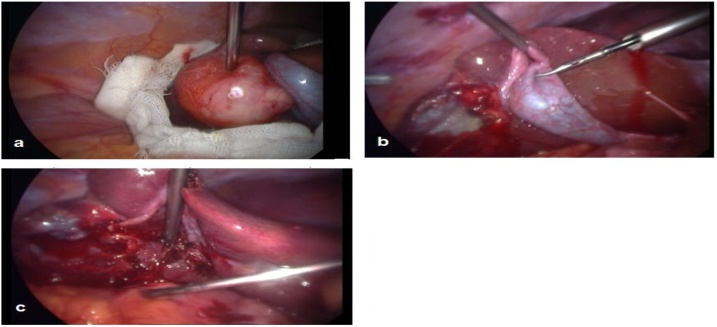
Laparoscopic surgery shows: a: Hydatid cyst. b: Cavity of the cyst after pericystectomy. c: Lower liver surface after cholecystectomy.

First of all, we started by introducing into the abdominal cavity a gauze soaked with hypertonic saline solution (10%) which was placed on the diaphragmatic surface of the liver around the cysts to prevent the spillage of fluid. Then we performed the decompression of the cyst by aspiration of the cyst fluid using a wide bore needle through the 5 mm port under laparoscopic guidance, taking care to avoid spillage, and by using a continuous suction cannula around the needle puncture site. We injected the hypertonic saline solution (10%) into the cyst cavity. Ten minutes later, we sucked it and opened the cyst using the electrocautery making a direct inspection of the cavity by introducing the laparoscope into the cyst to seek for remaining cyst elements and biliary leakage, if any, for subsequent attention. We performed then the lagrot partial pericystectomy for the hydatid cyst’s anterior wall, as much as possible, using diathermy and bringing it out of the abdomen. After a clear vision of the gallbladder, a standard cholecystectomy was performed from the same ports, and a drain was inserted under the liver that is removed later after 24 h when no signs of bleeding or biliary leakage were obtained. The patient was discharged in two days. The follow‐up was made as an outpatient at the 30th, 60th and 90th days postoperatively; the patient denied any symptom complaint or sign. [see Fig. 2].

## 4 Conclusion

The simultaneous of laparoscopic partial pericystectomy with other producers in the same surgical field may be safe in certain selective cases. Our report shows that these combined surgeries will not increase the risk of intra- or post- operative complications like surgical site infection, spillage or even the recurrence.

## 5 Discussion

In the management of abdominal hydatid cyst disease, several surgical techniques have been advocated, ranging from aspiration to radical and segmental resection. The surgical treatment of liver hydatid disease has evolved dramatically with the improved of the laparoscopy [1].

However, some manuscripts in the first decade of 21 st century reported an anaphylactic shock during the minimally invasive procedure of the hydatid cyst management; this strongly exaggerated the fear which seemed to discourage many surgeons from readily adopting these techniques [7,8]. Later, many publications put up rules and conditions in the laparoscopic approach: The deep-seated cysts; posteriorly located cysts (segments I, VII and VIII); and cysts characterized by Gharbi Classification as a type 4 or type 5 [9], all these situations would prevent the laparoscopic approach [10].

In general, the combined actions in one laparoscopy were discussed separately many times in selective cases [11]. The partial pericystectomy with total cystectomy for hydatid liver cysts were recently described in details as one laparoscopic surgery [6]; this approach did not show a higher risk or much difficulty in the prevention of hydatid spillage, sterilization and evacuation of the parasite or in the management of the residual cavity.

In our case we continue the discussion and questions that others began above; the abdominal hydatid cysts could be present with other pathological problems like a cholelithiasis. The cons and pros of combined surgery should be evaluated every time; the difficulty of techniques, risk of contamination, duration of the procedure and predicting of early or late complications play the main role in the decision of choosing either combined or separated interventions.

During the planned procedure; every technique that prevents spillage was done; every instrument that avoids the direct contact between the contents of the cyst and the abdominal cavity on one side; and between these contents and ports in another side were used. Then, the gallbladder was resected classically and the procedure was done, the patient then kept following-up till the complete healing and no residual or new hydatid cyst was reported that go on with perspective exhausted patient from repetition of surgeries.

## Conflicts of interest

We don't have any conflicts of interest with any organisation, on the contrary our case report was supported by three big educational institution.

## Sources of funding

We haven't received any funding for our manuscript because it is a case report not a research.

## Ethical approval

Our case is exempt from ethical approval in our institute.

## Consent

Written informed consent was obtained from the patient for publication of this case report and accompanying images. A copy of the written consent is available for review by the Editor-in-Chief of this journal on request [12].

## Author contribution

Aliaa Bakr: corresponding author/ collected the data and wrote the manuscript.

Mhd Belal Alsabek: co-author/ the surgeon who prepared the patient, planed the surgery, run the operation and reviewed the PubMed Library.

Abdul Rahman Hammadieh: supervisor and guarantor/ reviewed the manuscript.

## Registration of research studies

Our manuscript is a case report not a research.

## Guarantor

Abdul Rahman Hammadieh: MD, AFSA. Assistant professor in the Surgery Department, Damascus University, Faculty of Medicine, Damascus, Syria. The Head of General Surgery Ward, Al-Mouwasat University Hospital, Damascus, Syria.

## Provenance and peer review

Not commissioned, externally peer-reviewed.
